
JOURNAL INFORMATION
==============================
NLM Title Abbreviation: ScientificWorldJournal
Journal ID: ScientificWorldJournal
NLM Journal ID: 101131163
Journal ID: TSWJ

The Scientific World Journal

ISSN: 2356-6140
EISSN: 1537-744X
Publisher: Wiley

ARTICLE INFORMATION
==============================
PMCID: PMC6083890
PMID: 30147556
DOI: 10.1100/tsw.2001.114
Article ID: 190154
Article version: 1
Subjects: Short Report

Mechanisms Involved in the Enhanced Susceptibility of Senescent Rats to the Hepatocarcinogenic Effect of Peroxisome Proliferators: Role of Peroxisome Proliferator-Activated Receptor alpha (PPAR), Cell Proliferation and Oxidative Stress

Youssef Jihan A. 1
Ammann Pierre 2
Ghanayem Burhan L. 2
Birnbaum Linda S. 3
Badr Mostafa Z. 1 *
1 University of Missouri-Kansas City, Kansas City, MO 64108, USA
2 National Institute of Environmental Health Sciences, Research Triangle Park, NC 27709, USA
3 US Environmental Protection Agency, Research Triangle Park, NC 27709, USA
*Mostafa Z. Badr: badrm@umkc.edu
Publication date: 2001
Electronic publication date: 2001 Sep 24
Volume: 1
First page: 85
Last page: 85
Copyright: Copyright © 2001 Jihan A. Youssef et al.
Copyright year: 2001
License: This is an open access article distributed under the Creative Commons Attribution License, which permits unrestricted use, distribution, and reproduction in any medium, provided the original work is properly cited.
License URL: https://creativecommons.org/licenses/by/3.0/

Funding: National Institutes of Health CA/OD 74384 National Institutes of Health AG 18479

==============================
Miami *Nature Biotechnology* Short Reports
*TheScientificWorld* (2001) 1(S3), 85SR
ISSN 1532-2246; DOI 10.1100/TSW.2001.114

### MECHANISMS INVOLVED IN THE ENHANCED SUSCEPTIBILITY OF SENESCENT RATS TO THE HEPATOCARCINOGENIC EFFECT OF PEROXISOME PROLIFERATORS: ROLE OF PEROXISOME PROLIFERATOR-ACTIVATED RECEPTOR *ALPHA* (PPARα), CELL PROLIFERATION AND OXIDATIVE STRESS

Jihan A. Youssef[1], Pierre Ammann[2], Burhan L. Ghanayem[2], Linda S. Birnbaum[3] and Mostafa Z. Badr[1,*]

[1]University of Missouri-Kansas City, Kansas City, MO 64108, [2]National Institute of Environmental Health Sciences, Research Triangle Park, NC 27709 and [3]US Environmental Protection Agency, Research Triangle Park, NC 27709
[*] Badrm@UMKC.edu

**INTRODUCTION.** Nongenotoxic peroxisome proliferating chemicals (PPs) cause hepatocarcinogenesis in rodents, with senescent animals being more susceptible than their younger counterparts (1). Theories have been advanced suggesting a PPARα-dependent role involving Kupffer cells, growth regulating genes, or oxidative stress in PP-induced hepatocarcinogenecity. The purpose of this study was to investigate mechanisms involved in the enhanced susceptibility of senescent animals to these chemicals.

**METHOD.** Using RT-PCR, we investigated age-dependent hepatic gene expression of PPARα, proliferating cell nuclear antigen (PCNA), and the $H_2O_2$-producing peroxisomal enzyme acyl-CoA Oxidase (Acose) in male Fischer-344 rats of 4, 10, 50 and 100 weeks of age. PPs used were: Diethylhexylphthalate (DEHP, 1.2 g/kg), Wy-14,643 (250 mg/kg), and clofibrate (250 mg/kg), given orally in 1 ml corn oil. Control animals received the corn oil vehicle. All animals were sacrificed 48 hrs post dose.

**RESULTS.** DEHP, and Wy-14,643 increased liver/body weight ratios more dramatically in young animals (4 weeks old), than in all other age groups within 48 hrs. Clofibrate did not cause hepatomegaly in any group. Despite a significant difference in the hepatocarcinogenic potency of Wy-14,643 and DEHP (2), these two PPs increased hepatic expression of PCNA to comparable levels in all age groups. Ironically, clofibrate which is a weak hepatocarcinogen (2) increased PCNA mRNA expression to significantly higher levels in 4-week-old rats compared to 100-week-old rats. In addition, Wy-14,643 enhanced the expression of Acose mRNA to comparable levels in all age groups, while clofibrate increased the expression of this gene to higher levels in young, adult rats (10 weeks old), than in senescent rats (100 weeks old).

**DISCUSSION.** Data suggest that hepatic levels of PPARα gene expression, cell proliferation, or oxidative stress do not adequately explain the known enhanced susceptibility of the aged liver to PP-induced hepatocarcinogenecity. This conclusion is supported by our findings showing that age-related changes in these parameters, in response to treatment with PPs, did not correspond with the known rank of hepatocarcinogenic potencies of the tested chemicals.

Other factors such as the impact of aging on PP-induced inhibition of hepatocellular apoptosis remain to be evaluated.

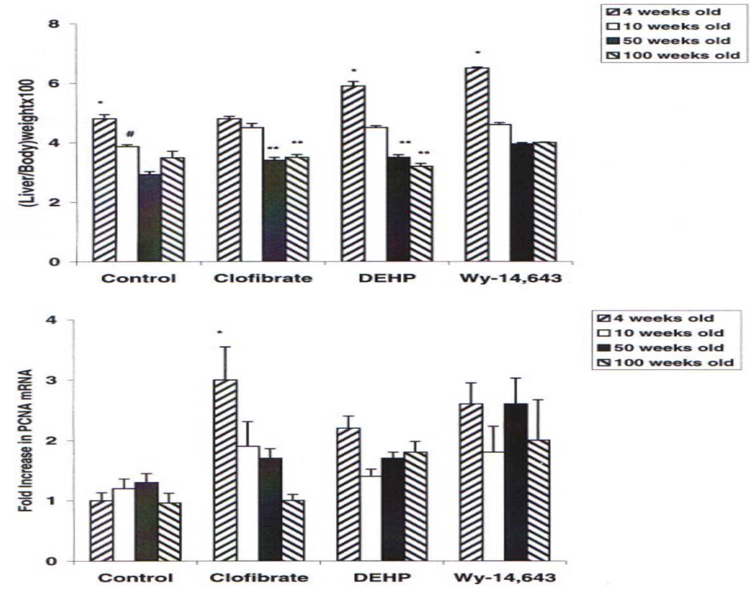

**ACKNOWLEDGEMENTS.** This work was supported by NIH grants CA/OD 74384 and AG 18479; this abstract does not necessarily reflect EPA policy.

**REFERENCES.**

1. Youssef, J. and Badr, M. (1999) Environ. Health Perspect. 107, 791-797
2. Youssef, J. and Badr, M. (1998) CRC Crit. Rev. Toxicol. 28, 1-33
